# An in vitro comparison of root canal system prepared with either hand or rotary instruments 

**Published:** 2010-11-15

**Authors:** Azza A Dafalla, Neamat Hassan Abubakr, Yahia E Ibrahim

**Affiliations:** 1 *Department of Conservative Dentistry, School of Dentistry, Africa University of Medical Science, Khartoum, Sudan*; 2 *Department Professor of Conservative Dentistry, Faculty of Dentistry, University of Khartoum, Khartoum, Sudan *; 3 *Department of Conservative Dentistry, Faculty of Dentistry, University of Khartoum, Khartoum, Sudan*

**Keywords:** Root canal preparation, K-file, NiTi rotary files, permanent teeth

## Abstract

**Introduction:** The aim of the present study was to compare hand stainless steel K-files and Nickel-Titanium Profile 0.04 taper 29 series rotary instruments for their efficiency, procedural errors and time consumed in preparation of root canal system.

**Materials and Methods:** A total of 46 maxillary and mandibular first premolars extracted for orthodontic purposes were collected (two contralateral teeth from each individual). The samples were divided into two groups of 34 canals each. Teeth in the first group were prepared with stainless steel hand K-files while the second groups were prepared with profile 0.04 taper series 29 rotary files. Preparation period was recorded for both groups. Impression material was introduced into the prepared canals so that the replica of prepared canals was achieved. These were assessed under stereomicroscope to assess the efficiency in preparing canals in respect to canal smoothness, ability of impression material to flow and quality of taper.Statistical analyses were performed using t-test, Chi-square and Fishers exact tests.

**Results:** Results showed significantly shorter preparation time for Profile than K-file. 8.8% of the canals prepared with K-files showed canal blockage, while all canals prepared with Profile remained patent. Alterations in working length working distance appeared in 23.5% of canals prepared with K-file and 11.7% in canals prepared with Profile. Failed instruments in K-files were significantly higher, mostly deformation (P<0.001). Profiles failed instruments were in the form of fracture and no deformation was detected. Both systems showed unsatisfactory walls smoothness and flow.

**Conclusion:** Within the limitation of this study it was concluded that Profile 0.04 taper series 29 rotary systems prepare canals more rapidly, and have lower incidences of fracture and blockages, and only limited loss of working length. Canal preparation with K-file was time consuming and showed higher incidence of deformed instruments; overall, rotary instruments seem to offer greater advantages.

## Introduction

The technical demands and level of precision required for successful performance of endodontic procedures have traditionally been achieved by careful manipulation of hand instruments within the root canal space and by strict adherence to the biologic and surgical principles, essential for disinfection and healing (1). To improve the speed and efficiency of the treatment stainless steel instruments have been used in a variety of preparation techniques, in an attempt to produce the appropriate canal shape. However, studies have shown that procedural incidents occur commonly, producing aberrations such as formation of hourglass-shaped canals, zips, elbows and canal transportation (2-4). Nickel-Titanium (NiTi) rotary instruments are thought to reduce such aberrations. 

Furthermore, NiTi instruments maintain the original canal shape during preparation and have a reduced tendency to transport the apical foramen (5-7). With all these apparent advantages, the use of NiTi rotary systems has increased considerably since their introduction. However, their cost, instrument fracture (8-10) and their tendency to straighten in severely curved canals leading to loss of original canal shape (11-14) are notable disadvantages. Although few studies have been carried out into the shaping ability of rotary NiTi files, they have been shown to be faster than hand preparation, potentially reducing patient and dentist fatigue (11,15,16). 

Recently there had been a total shift from manual root canals preparation to rotary instrumentation due to its accuracy and shorter preparation time. On the other hand, the conventional hand instruments are still commonly used for canals preparation in dental schools and general dental practices. The aims of the present study are to assess efficiency of one of the rotary instruments and to compare it with hand stainless steel K-files for their efficiency, procedural errors and time consumed in preparation of root canal system.

## Materials and Methods

Bilaterally premolars extracted for orthodontic reason were collected and immediately stored in 10% formalin solution (17,18).The teeth were with complete apex formation and showed total of 68 canals. Canals curvature was measured according to Schneider (19). It measures the degree of the curvature in order to categorize root canals as straight (5Â curvature or less), moderately (10-20Â) or severely curved (>20Â). Samples were randomly divided into two experimental groups. Each group had a total of 34 canals. Group 1 was prepared by conventional methods with stainless steel hand K files (Mani, Japan) and group 2 with the rotary method using NiTi Profile 0.04 taper 29 series (Dentsply, Tulsa Dental, Tulsa, OK, USA). All teeth were embedded in acrylic resin blocks (Egypharma, Cairo, Egypt) keeping the apex out of the resin. Access cavities were prepared; working length (WL) of each canal was determined by introducing a size 10 K-file into the canal until it was just visible at the apical foramen; WL was taken 1mm short of this point. Subsequently, instrumentation was performed. For canal preparation in group 1, modified double flare technique by stainless steel K-files was used. Instrumentation started with size 15 file then enlarged to size 45 as the master apical file. Copious irrigation with 2.5% NaOCl (hyposol, ST Decon Labs, Inc, USA) was used throughout the preparation and patency was maintained in all the canals by recapitulation using a size 15 K file.

In group 2, teeth were instrumented with profile 0.04 taper 29 series rotary instruments. The instruments were used according to manufacturer instructions in a torque controlled motor and handpiece (Endo-Mate DT. NSK, Japan). The Profile instruments were used in a crown-down technique. Commencing with a size 4 Profile 0.04 Taper, the instrument was used at 280-300 rpm with a slow apical progression to 1/2-2/3 of the estimated length of the canal. The process was repeated with a files size 5, 6 and 7. A size 3 file was then used to reach 2/3-3/4 of the estimated length of the canal. The definitive WL was then determined with a size 10 K-Flex file. A size 3 file was used to reach the WL and the canal was sequentially enlarged so that a size 7 file reached the WL. The definitive WL was then determined with a size 10 K-Flex file. Copious irrigation was used throughout the procedure with 2.5% NaOCl. Patency filing was checked at the end of the instrumentation procedure. In all groups a new set of files was used to instrument five teeth and then discarded. 

The internal three-dimensional shape of all canals was determined from intracanal impressions. A small amount of canal lubricant, RC-prep (Premier Products Co. USA) was introduced into the canal lumen. Light bodied condensation silicone impression material (Oranwash Zhermack, Badia Polesine, Italy), was injected carefully into each canal, and followed by the introduction of a fine barbed broach (Mani, Utsunomiya, Tochigi***,*** Japan) to act as a support for the 

**Figure1:**
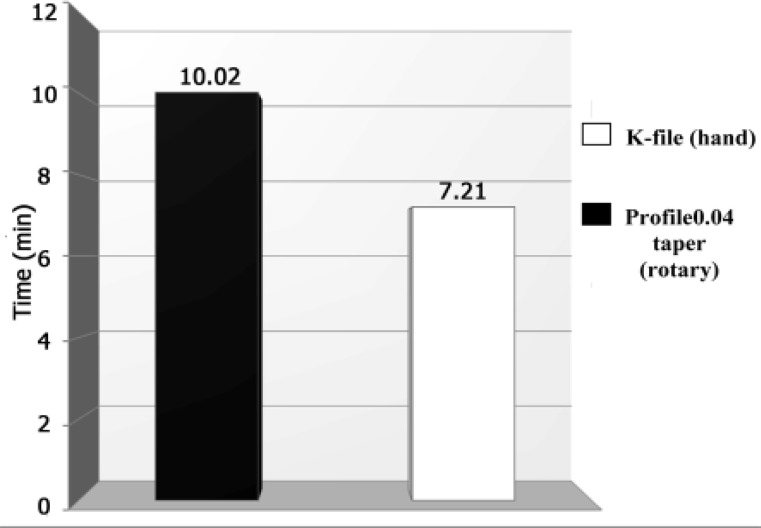
Comparison of canals’ means preparation time between K-file and profile 0.04 taper (rotary)

**Figure3:**
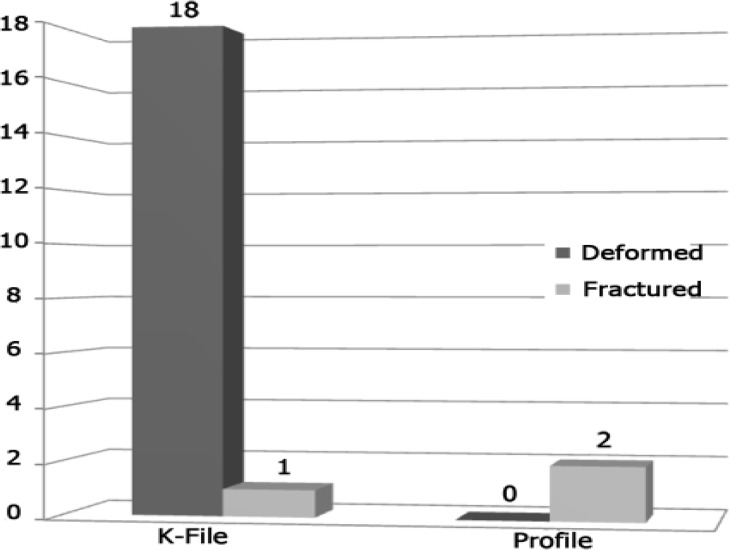
Final canal impressions for single rooted and double rooted first premolars

coronal part of the impression and to facilitate removal. The impressions of the prepared canals were removed and assessed under the ×40 magnification of stereomicroscope (Micros, Austria) using Abou-Rass and Jastrab criteria (20) (Table 1).

Other factors were also recorded *i.e.* preparation time, canal blockage, loss of working distance and instruments failure.

Software of Statistical Package for Social Sciences (SPSS) was used to analyze the data at a confidence level of 95%, using unpaired t-test for preparation time analysis, Chi square and Fishers exact tests for canal form analysis. Differences were considered significant when the probabilities were equal or less than 0.05.

## Results

***Preparation time:***

There was a significant difference between the two methods in term of preparation time (Figure 1). Canal preparation time measured in minutes and seconds showed a mean time of 10:02±3:34 SD for hand K-files preparations and mean time of 07:21±3:04 SD for rotary Profile preparations, which was statistically significant (P=0.02). 

**Figure2:**
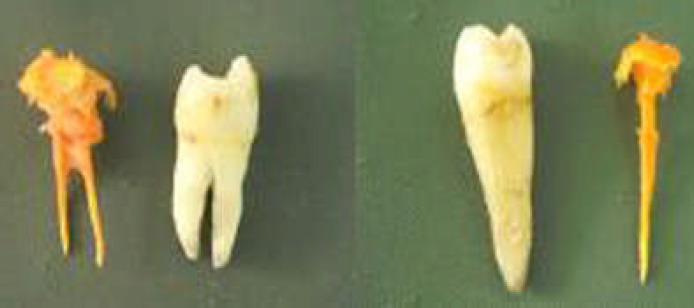
Comparison of instruments failure between K-file and Profile in canals reparation

***Canal blockage:***

In hand prepared canals using K-file the results have shown that 3 canals (8.8%) were blocked

by debris. Following rotary instrumentation with profile system, all the 34 canals remained patent (P=0.11) (Table 2). 

***Change of working distance:***

In hand preparation method, eight canals (23.5%) were associated with loss of working length due to canal blockage. 

***Instruments failure:***

Hand preparations with K-file showed total of 19 failed instruments, 18 of them (94.7%) were permanently deformed and only one instrument (5.3%) was fractured. Rotary preparation with Profile system reported two canals with instruments fracture (failure) of files size 3 and size 5. These two canals showed curvature of 40^°^ at 8mm from the orifice. No deformed instrument was reported (Figure 2). Hand instrumentation showed 90.5% failure (18 files deformed) compared to 9.5% (2 files fractured) failure occurred in Profile 0.04 file. The difference was highly significant (P<0.001).

***Canal form:***

Quality of prepared canal was assessed from the intracanal impression (Figure 3). Table 3, showed the quality of the apical stop, smoothness of the canal; together with the presence and absence of vertical grooves, flow and taper of the assessed canals.

**Table1:** Categorization of canal form

**Categorization**		**Canal form**
Absent, poorly defined, well defined	**Apical stop**	
Poor, good	**Apical half smoothness **	
Poor, good	**Coronal half smoothness **	
Absent, present	**Horizontal/longitudinal grooves**	
Good (continuous blending of the canal from orifice to apical stop), poor (abrupt changes in direction and the presence of ledges)	**Flow**	
Good (conical shape), poor (hourglass or cylindrical shapes)	**Taper**	

**Table2:** Canal blockage with debris following instrumentations with K-files and Profiles

Canal status	K-file	Profiles
Blocked	**3(8.8%)**	**0**
Patent	**31(91.2%)**	**34(100%)**
Total	**34**	**34**

## Discussion

Bilaterally extracted humans first permanent premolars were used in this study. Previous studies used simulated canals constructed in clear resin block with standardization of degree, location and radius of root canal curvature, this guaranteed high degree of reproducibility and standardization of the experimental design in assessment of with K-files and Profiles prepared canals preparation procedures and instruments performance. However, regarding micro-hardness and abrasiveness of acrylic resin when compared to dentin, it has been expressed that dentin usually requires double the preparation forces (21,22). In this study, extracted human teeth were used to simulate the clinical situation. The ProFile series has been set as the gold standard (23) of NiTi rotary instruments. Some investigations have reported that rotary NiTi instruments do not clean root canal walls effectively, particularly the apical part of curved canals (24,25). Additional concern has been expressed about the comparatively high incidence of fractures in rotary NiTi instruments (26). On the other hand, shaping of curved canals with stainless steel K-files manipulated in a linear filling motion proved a satisfactory method to maintain the original canal curvature (27).

Stainless steel files were well known for creating aberrations in canals (28), but it appeared that this was the result of their inherent metal stiffness which is confounded by instruments design and canal shape (24,29).

**Table 3:** Assessment of canal form from intracanal impression

**P-value**	**Profiles**	**K-file**	**Category**
**0.38 **	**14 (41.2%)**	**10 (29.5%)**	Apical stop
**0.310 **	**15(44.1%)**	**12(35.3%)**	Apical smoothness
**0.308**	**33(67.7%)**	**20(58.8%)**	Coronal smoothness
**0.5 **	**19(56%)**	**18(53%)**	Horizontal and vertical grooves
**0.50 **	**17(50%)**	**18(53%)**	Flow
**0.044 **	**14 (41.2%)**	**22(64.8%)**	Taper

 In the present study, the time taken to prepare the canals with K-file was significantly longer than that taken by the 0.04 taper Profile. Many studies reported more rapid preparation for rotary instruments than hand ones (13,30,31);

while other studies have shown no difference (32-35). It is likely that working time is more dependent on operator factors and the used preparation technique rather than the instruments themselves. Mesgouez *et al.* reported that time required for canal preparation with Profile was inversely related to operator experience; the inexperienced operator demonstrated a significant linear regression between canal number and preparation time (36). Overall, NiTi rotary preparation is efficient to reduce patient and operator fatigue whilst providing safe handling of instruments in the handpiece (37). Stainless steel K-files showed significantly higher incidences of instruments failure. This high deformity may be due to the low modulus of elasticity of the material that makes it bend within canals (31). The Profile instruments showed failure in the form of fracture; only two instruments were fractured without prior evidence of plastic deformation at the fifth use (38). Findings in this study were in contrast to Al-Omari *et al.* who reported that the larger Profiles size were associated with more deformation contrary to what was noted with stainless steel files (39). However, the low incidence of profile instruments fractures was also reported by Defoire *et al.* who suggested its continuous use in root canal treatment even by dental students in laboratories, if preventive methods are used (40,41). Yared *et al*. demonstrated that ProFile 0.04 instruments were safe to be used without any fracture with low-torque and high-torque motors (42).

This study showed an overall change in working distance in both samples prepared with K-file hand instruments and Profile rotary instruments and it was noted that loss of working distance was associated with increased canal curvature. Al-Omari *et al.* reported that with stainless steel hand instruments, length was lost following combination of canal blockage, straightening of the canal and/or the creation of aberrations such as ledges (43). This does not appear to be a common problem with NiTi rotary instruments as, despite potential problems with operator control, there appears to be only limited change (11,13,37) mostly in the form of over instrumentation and increase in working distance as a result of lack of tactile sensation, certainly this was the case in this study.

This study demonstrated that canals prepared with Profiles showed better quality of apical and coronal smoothness than those prepared with K-files; concurring with Thompson and Dummer (37). Interestingly, there was a significant difference in canal taper between the two methods; stainless steel K-files showed better taper qualities than profiles in prepared canals. This may be due to previous reports that rotary files tend to create slight canal transportation toward the outer aspect of the curvature in the apical region of the canals (12,35,44). On the other hand, two reports contradict the results of taper quality of Profiles in this study; Nagratna reported that good taper quality was more significant using Profiles (31). Thompson reported that Profiles produced tapered preparation in all of the specimens prepared in study and positive characteristics are presumably a reflection of their planing action during rotation; such a canal shape would appear to facilitate obturation (44).

## Conclusion

Profile 0.04 series 29 rotary systems prepare canals more rapidly, and showed low incidences of blockage, and only limited loss of working length. Canal preparation with K-file was time consuming and showed higher incidence of deformed instruments probably due to low elasticity of the stainless steel metal. Both systems showed unsatisfactory results for canal walls smoothness and flow, but K-files showed better canal tapering.
